# Effect of DMSO on lifespan and physiology in *C. elegans* : Implications for use of DMSO as a solvent for compound delivery

**DOI:** 10.17912/micropub.biology.000634

**Published:** 2022-09-07

**Authors:** Abdelrahman AlOkda, Jeremy M. Van Raamsdonk

**Affiliations:** 1 Department of Neurology and Neurosurgery, McGill University, Montreal, Quebec, Canada; 2 Metabolic Disorders and Complications Program, and Brain Repair and Integrative Neuroscience Program, Research Institute of the McGill University Health Centre, Montreal, Quebec, Canada; 3 Division of Experimental Medicine, Department of Medicine, McGill University, Montreal, Quebec, Canada

## Abstract

Dimethyl sulfoxide (DMSO) is a solvent that has been used for basic and medical research based on its ability to dissolve both polar and non-polar compounds. In order to use DMSO to deliver compounds that may impact longevity or neurodegeneration, it is important to first determine the effects of DMSO on aging and physiology. We examined the effect of different concentrations of DMSO on lifespan and development time in *C. elegans.* We found that DMSO concentrations up to 2% DMSO did not affect longevity in wild-type worms, while concentrations of up to 0.5% DMSO were compatible with normal development times. 0.5% DMSO also had minimal effect on fertility and movement. In summary, our results show that concentrations of DMSO up to 0.5% can be safely used to deliver compounds to *C. elegans* with little or no modifying effects on lifespan or physiologic rates.

**Figure 1. Effect of DMSO on longevity and physiologic rates:**
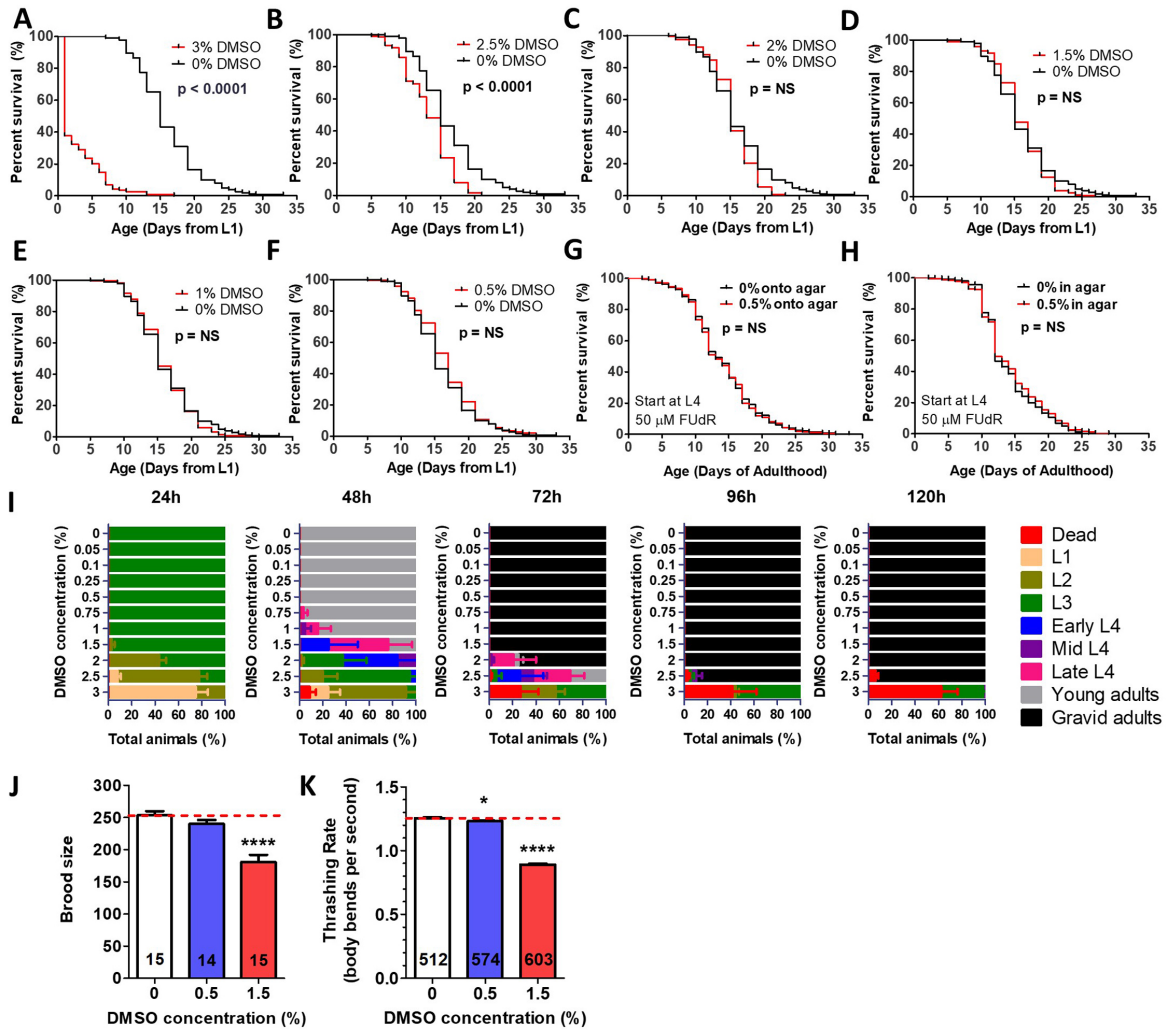
In order to use DMSO as a solvent for compound delivery, the effect of different concentrations of DMSO on aging and physiology was examined. DMSO concentrations of 3% ( **A** ) or 2.5% ( **B** ) resulted in a significant decrease in lifespan. In contrast, DMSO concentrations of 2% or less had no effect on longevity ( **C-F, extended data** ). For panels A-F, exposure to DMSO was initiated at the L1 stage and DMSO was added on top of the plate as described in the methods section. The results are pooled from four biological replicates and have at least 114 animals per condition. At a concentration of 0.5% DMSO, when DMSO exposure was initiated at the L4 stage on plates containing 50 µM FUdR, lifespan was not affected whether the DMSO was added on top of the plate ( **G** ), or in the plate during plate pouring ( **H** ). The results from panels G and H are pooled from three biological replicates and have at least 776 animals per condition. Significance of all lifespan experiments was determined using the log-rank test. When synchronized L1 larvae were grown on NGM plates containing DMSO at different concentrations, DMSO concentrations of up to 0.5% resulted in normal post-embryonic development times, while concentrations of 0.75% and above resulted in developmental delay ( **I** ). There was minimal effect of 0.5% DMSO on either brood size ( **J** ) or thrashing rate ( **K** ), while 1.5% DMSO resulted in a significant decrease in both phenotypes. Sample sizes ( *n* numbers) are indicated in the bars of each graph. Bars indicates the sample mean. Errors bars show the standard error of the mean (SEM). The p-value was determined using a one-way ANOVA with a Dunnett's Multiple Comparison Test. * *p* < 0.05; **** *p* < 0.0001 . All assays were performed at 20 ^o^ C.

## Description

Dimethyl sulfoxide (DMSO) is frequently used as a solvent in biological studies and as a vehicle for compound delivery due to its amphipathic properties and chemical stability (Cheng et al., 2003; Santos et al., 2003). Indeed, most of the commercially available compound libraries have been created with DMSO as the solvent. In choosing a solvent to use for our experiments with *C. elegans,* we sought to determine the maximum concentration of DMSO that could be utilized without affecting worm health as measured by lifespan and physiologic rates. In reviewing the literature, we found variable results with different studies reporting that DMSO can increase (Frankowski et al., 2013; Goldstein et al., 1992; Wang et al., 2010), decrease (Solis & Petrascheck, 2011), or not affect (Frankowski et al., 2013; Zwirchmayr et al., 2020) the lifespan of *C. elegans* . These differences suggest that the exact experimental conditions may determine whether or not DMSO has an impact on worm health.

In this work, we investigated the effect of DMSO on lifespan, development time, fertility and movement using a method of administration that we developed for chemically sensitive compounds, which may be disrupted when added to warm nematode growth medium (NGM) agar prior to pouring. In essence, this approach involves diluting a stock solution of the chemical compound dissolved in DMSO to 10X of the final desired concentration in water, then adding the compound on top of standardized NGM plates seeded with OP50 bacteria (food source) to dilute to 1X and allowing the plates to equilibrate overnight before introducing the animals. To explore the effects of DMSO, we raised wild-type N2 animals on NGM plates with DMSO administrated on top of the plate at dosages ranging from 0.05% to 3% final volume ratio.

We found that exposure to DMSO beginning at the L1 stage of development significantly decreased mean lifespan at concentrations of 3% and 2.5% but had no effect at concentrations of 2% and less ( **Figure 1A-F, extended data** ). In contrast to our findings, a previous study reported lifespan extension with DMSO concentrations ranging from 0.01% to 2%, and shortened lifespan at 5% DMSO using a different administration paradigm (Wang et al., 2010). The main differences between our protocol and the previous study were that Wang et al. added DMSO directly into the agar during plate pouring, FUdR was included in the lifespan assay to prevent progeny production and exposure to DMSO was begun at the L4 stage. To determine whether one of these factors could account for the differences in the outcome, we repeated the lifespan experiment using FUdR and beginning DMSO exposure at the L4 stage. However, whether we added the DMSO onto the agar ( **Figure 1G** ) or included DMSO in the agar during pouring ( **Figure 1H** ), we found that DMSO did not increase lifespan at a 0.5% concentration, the concentration at which Wang et al. observed the maximum lifespan extension.

Next, we examined the effect of DMSO administration on three additional measures of worm health: post-embryonic development, reproductive fecundity, and movement in liquid (thrashing rate). We found that worms grown on DMSO concentrations of 0.5% or less developed at the same rate as control worms on 0% DMSO, while higher concentrations resulted in a dose-dependent developmental delay ( **Figure 1I** ). A previous study reported that DMSO concentrations of 0.06% can lead to increased post-embryonic developmental time (Xiong et al., 2017).

In examining the effects of DMSO on fertility, we found that 0.5% DMSO did not significantly affect brood size, while a higher concentration of 1.5% DMSO resulted in decreased brood size ( **Figure 1J** ). Similarly, exposure to 0.5% DMSO resulted in a small, less than 2% decrease in thrashing rate, while 1.5% DMSO decreased thrashing rate by 29% ( **Figure 1K** ). In agreement with our results, Wang et al. and Boyd et al. found that 0.5% DMSO did not affect brood size, however, Goldstein et al. reported a significant decrease in the overall worm population grown on 0.5% DMSO (Boyd et al., 2010; Goldstein et al., 1992; Wang et al., 2010). It has also been reported that DMSO decreases pharyngeal pumping at concentrations greater than or equal to 0.25% (Calahorro et al., 2021; Raizen et al., 2012).

In practice, the selection of solvents as vehicles requires surveying of the literature for their reported biological activities that might interfere with the experimental outcome. For example, ethanol or acetone may be more suitable for dissolving compounds for use in pharyngeal pumping studies in *C. elegans* as DMSO at concentrations of 0.25% and above can inhibit pharyngeal pumping (Calahorro et al., 2021; Raizen et al., 2012). For longevity studies on the other hand, treatment with ethanol at 0.5% and 1% from egg or young adulthood stage was shown to increase lifespan of *C. elegans* (Bilodeau, 2021). Despite that, developmental exposure to ethanol at 0.2 M (which equivalates roughly to 1% ethanol v/v%) was reported to significantly decrease lifespan and pharyngeal pumping (Davis et al., 2008). Together, the inconsistency in the literature regarding solvent vehicle effects begs the question of what factors contribute to such observations.

Chemical dynamics and kinetics govern how the solvents behave in the system (i.e., in liquid medium, added in a solid agar, etc). For instance, NGM plates prepared with highly volatile solvents such as ethanol during the pouring step can have concentration variability due to ethanol evaporation. On the other hand, adding the solvent in a concentrated form directly to the agar surface requires time to allow the solvent to diffuse and equilibrate throughout the medium. In addition, the final solvent concentration reaching the worms, and the associated effects respectively, may be affected by the amount of biomass that is present through dilution, depletion, metabolism or shielding. It is possible that the individual exposure to solvent in worms cultured at higher worm and/or bacterial culture densities is lower compared with worms grown at lower culture densities. Furthermore, *Escherichia coli* can metabolize DMSO to dimethyl sulfide (DMS) catalyzed by their DMSO reductase enzyme causing the overall DMSO depletion (Sambasivarao & Weiner, 1991). Thus, one needs to also keep in mind the potential effects of solvent metabolic byproducts. This includes the bioconversion of DMSO to dimethyl sulfone (DMSO _2_ , also known as methylsulfonylmethane or MSM) in the presence of oxygen (He & Slupsky, 2014). Indeed, Goldstein et al. showed that worms exposed to DMSO _2_ exhibits greater abnormalities on lifespan, developmental time and fecundity compared to DMSO at the same given concentration (Goldstein et al., 1992).

Overall, our results demonstrate that DMSO at concentrations of up to 0.5% can be safely used to deliver compounds with minimal effect on *C. elegans* lifespan or physiologic rates. Nonetheless, as the precise method of DMSO administration can impact the outcome, it is important to examine the effects of DMSO administration using the paradigm that will be used in a given experiment to identify a concentration that will not affect worm physiology and lifespan. Importantly, this work draws attention to the importance of evaluating the effects of solvents on organismal physiology in order to minimize the effects of the solvent on the outcome of the experiment.

## Methods

***C. elegans strains*** . N2 (wild-type) worms were used for this study. Worms were grown at 20 °C on 60 mm NGM-agar plates spotted with *Escherichia coli* OP50 bacteria. For all of the experiments below, worms were synchronized by bleaching and thereafter allowed to hatch for 24 h at 20 °C in a 15-mL centrifuge tube on a rotating wheel.

***Bacteria preparation.*** A single *E. coli* OP50 colony was inoculated in 250 mL LB medium in a 1 L Erlenmeyer flask and incubated for 20 h at 37 °C with 250 RPM shaking. The bacteria were collected in a 50-mL centrifuge tubes and centrifuged at 3900 g for 10 minutes before discarding half of the supernatant and resuspending the pellet. The OD600 of the concentrated culture was typically between 6.45 and 6.55 (equivalent to 5.16 x 10 ^9^ – 5.24 x 10 ^9^ CFU/mL).

***Plate preparation.*** For all experiments, vented 35 mm plates were utilized. Autoclaved NGM was added in a volume of 2.5 mL to each plate and allowed to dry overnight before spotting 250 µL of freshly prepared concentrated OP50 culture. The plates were allowed to dry for an additional 3 days before storing inverted in air-tight plastic boxes in the dark at 4 °C. The plates were utilized within 1 week of storage. For all the experiments, the treatment/control plates were prepared the day before utilizing. DMSO was diluted in 10X the final desired concentration in sterile ddH _2_ O and added in a volume of 250 µL per plate (e.g., for 1% v/v final DMSO plate, add 250 µL of 10% DMSO). The plates were allowed to soak the solution for 5-10 minutes lid-closed on a nutating mixer before sealing the plates with labelling tape and incubating inverted in a plastic box at 20 °C for 20-24 h to equilibrate.

***Lifespan* .** From L1 stage : Synchronized L1 worms were pipetted onto treatment or control plates. From L4 stage : Synchronized L1 worms were pipetted on control plates and allowed to grow until mid L4 stage before transferring them to treatment or control plates supplemented with 50 µM 5-Fluoro-2′-deoxyuridine (FUdR). Worms were transferred to new plates and scored for death every 1-3 days at approximately the same time. Total number of animals used in each biological replicate can be found in extended data.

***Post-embryonic development* .** Synchronized L1 worms were pipetted on treatment or control plates and were monitored every 24 h for their developmental progress. The results were pooled from four biological replicates and at least 20 animals were quantified per condition per replicate.

***Brood size.*** After reaching L4 stage on treatment or control plates, five worms were singled and moved to new treatment or control plates every 24 h until progeny production ceased. To facilitate offspring counting, the plates were stored at 4 °C once the progeny reached L4-young adulthood stage and were counted later after. The results were pooled from three biological replicates.

In all these methods, worms were censored if they were missing, have crawled off, have burrowed, or displayed internal hatching (matricide) or vulval rupture. However, these censored worms were still included in subsequent statistical analysis for lifespan.

***Thrashing* .** Synchronized L1 worms were pipetted on treatment or control plates and allowed to grow until the young adulthood stage. A volume of 1 mL of M9 buffer was added to the worms and they were left to acclimatize for 1 min before capturing 1 min video at 14 FPS using WormLab (MBF Bioscience). The videos were analyzed using wrMTrck plugin for the open-source image processing software, Fiji. Worm tracks were only considered for analysis when satisfied at least 420 frames (30 seconds). The results were pooled from three biological replicates.

***Statistical analysis* .** For lifespan experiments, GraphPad Prism 5 software was used to generate lifespan graphs and OASIS software (version 1) was used for statistical analysis to determine mean lifespan (Yang et al., 2011). P values were calculated using the log-rank (Mantel–Cox) method and Bonferroni correction was performed to account for the multiple comparisons. Otherwise, quantitative data were analyzed using GraphPad Prism 5. A one-way ANOVA was performed with a Dunnett's Test. A value of p < 0.05 was used to establish statistical significance and p-values are presented in the figure legend.

## Reagents

***Strains.*** N2 wild-type (Wild isolate from Bristol)

***Chemicals* .** Dimethyl sulfoxide (D8418, Sigma-Aldrich), 5-Fluoro-2′-deoxyuridine (F0503, Sigma-Aldrich)
